# Epithelial skin cancer before, during, and after the COVID-19 pandemic

**DOI:** 10.3389/fmed.2026.1833019

**Published:** 2026-08-05

**Authors:** Anna Pisarek, Robert Sabat, Kerstin Wolk, Sylke Schneider-Burrus

**Affiliations:** 1 Translational Skin Inflammation Research, Department of Dermatology, Venereology and Allergology, Charité—Universitätsmedizin Berlin, Corporate Member of Freie Universität Berlin and Humboldt- Universität zu Berlin, Berlin, Germany; 2 Centre for Dermatosurgery, Havelklinik, Berlin, Germany

**Keywords:** basal cell carcinoma, COVID-19, epidemiology, epithelial tumor, healthcare, non-melanoma skin cancer, NMSC, pandemic, squamous cell carcinoma

## Abstract

**Background:**

The COVID-19 pandemic caused significant changes in healthcare, but its effects on patients with severe skin diseases are not yet fully understood.

**Objectives:**

To assess the impact of the COVID-19 pandemic on characteristics of non-melanoma skin cancer in a German patient population.

**Methods:**

A retrospective single-center study analyzing data from patients who underwent surgical treatment for epithelial skin cancer before (Nov 2019–Feb 2020), during (Nov 2020–Feb 2021), and after the COVID-19 pandemic (Nov 2022–Feb 2023) at a Berlin hospital without drastic COVID-19-related care restrictions.

**Results:**

Data from 565 patients undergoing surgery for epithelial skin cancer were evaluated. Tumor thickness and the proportion of patients with more malignant tumors increased following the COVID-19 pandemic, while the proportion of patients with nodular basal cell carcinomas decreased. At the same time, the duration of inpatient stays decreased. Additionally, the proportion of patients with autoimmune diseases rose, placing a greater burden on the healthcare system and patients.

**Limitations:**

This was a retrospective single-center study of patients who had undergone elective surgery.

**Conclusion:**

These findings underscore the need for a sufficient number of specialized healthcare facilities to balance pandemic-related restrictions and new responsibilities with the continued treatment of serious illnesses.

## Introduction

In January 2020, the first patient in Germany fell ill with Coronavirus Disease 2019 (COVID-19), a highly infectious viral respiratory disease that was first reported in Wuhan, China, in December 2019 (1). As a result, not only contact limitations and lockdowns were imposed, but also access to the healthcare system was reduced and a large number of surgical procedures were postponed. In fact, from March 2020 onward, medical care in Germany was largely restricted to the treatment of life-threatening emergencies and patients with severe COVID-19 infections, especially during the first lockdown (2, 3). In numerous general hospitals, various specialized departments had to suspend their normal operations in order to treat patients infected with COVID-19. The increased exposure to infected patients raised the transmission risk among healthcare providers, resulting in higher staff absenteeism (4). New regulations had to be introduced to ensure prevention, treatment, and follow-up care of patients and the protection of staff. All of this led to an increased workload (5).

More than half of the participants in a structured survey of medical staff of dermatology departments in German hospitals reported that numerous elective procedures in their department had to be canceled at the onset of the COVID-19 pandemic (6). In contrast, essential interventions, particularly those involving malignant melanomas or large, rapidly growing, bleeding, or painful skin tumors could still be performed in most hospitals (6). Moreover, preventive services such as skin cancer screenings also declined significantly in Germany between 2020 and 2021 (7). Thus, colleagues from a dermatology practice in Bavaria reported an increase in tumor thickness of epithelial tumors during the COVID-19 pandemic (8). However, data on the impact of the pandemic on patients with such tumors who were treated in hospitals remain limited.

The aim of this study was to compare the features of patients with non-melanoma skin cancer (NMSC) and the characteristics of their tumors before, during, and after the pandemic in a hospital without drastic care restrictions. Our study focused on NMSC, as it is the most common malignant disease worldwide (9, 10). NMSCs include basal cell carcinoma and squamous cell carcinoma, with basal cell carcinoma being the most prevalent form of NMSC (9, 11). Squamous cell carcinoma and basosquamous carcinoma, a rare entity exhibiting features of both basal cell carcinoma and squamous cell carcinoma, are the most malignant forms of NMSCs (11, 12). In addition to basosquamous carcinoma, other subtypes of basal cell carcinoma include the less malignant nodular, sclerodermiform and superficial basal cell carcinomas as well as ulcerated variants (9, 11). Our specific goal was to identify potential associations between the COVID-19 pandemic and changes in the characteristics of epithelial tumors and care of patients with NMSC. As further pandemics are expected in the future, such findings could help make more informed decisions regarding the balance between pandemic-related restrictions in the healthcare system and maintaining access to medical care.

## Materials and methods

### Study design

We conducted a retrospective, single-center study analyzing data from patients undergoing surgery for epithelial skin tumors in the Center for Dermatosurgery at the Havelklinik, Berlin, Germany.

Three periods of time were examined: prior to the COVID-19 pandemic (Nov 2019 - Feb 2020; period 1), during the peak of the COVID-19 pandemic (Nov 2020 - Feb 2021; period 2) and after the peak of the pandemic (Nov 2022 - Feb 2023; period 3). The three periods are of equal length and cover the same calendar months to ensure comparability and eliminate seasonal effects. The period Nov 2020 - Feb 2021 coincides with a major wave of the pandemic and associated restrictions, making it the most appropriate timeframe for assessing the pandemic’s impact. The period Nov 2021 - Feb 2022 was not included because it represented a transitional phase with fluctuating restrictions and health conditions, which would have led to heterogeneity. Instead, we selected a later period (Nov 2022 - Feb 2023) to examine a more stable post-pandemic situation.

The Havelklinik, a hospital for elective orthopedic, vascular, abdominal and dermatological surgery, is visited by numerous patients from different social backgrounds due to its reputation and location on the outskirts of Berlin. The Havelklinik neither has an intensive care unit nor an emergency room; it was never part of the emergency plan during the early stage of the COVID-19 pandemic. Thus, surgical procedures in the clinic continued, apart from the complete lockdown, and the dermatological ward remained open for skin cancer treatment.

### Data collected

The following data were collected from electronic and paper-based sources: age at the time of surgery, length of hospital stay, gender, quantity of NMSC (basal cell carcinoma and squamous cell carcinoma), location of all tumors, location of the tumor with the highest thickness and further tumor-specific data (see below). For patients with multiple tumors, data from the thickest tumor were used for the evaluation of tumor type, thickness and location. If the thickest tumor was ulcerated, this was also noted. In addition, patients’ comorbidities, medication intake (immunosuppression) as well as alcohol and nicotine consumption were documented.

The following tumor-specific data from histopathological reports were collected: thickness in mm, horizontal extent in mm, number of excisions to achieve clear resection margins (R0) and ulceration. All histopathological evaluations were performed by the same dermatopathology unit, specifically by one of two highly experienced dermatopathologists. Additionally, for squamous cell carcinoma, the following parameters were collected: tumor stage (TNM classification) and tumor grading (G1-G4). For basal cell carcinoma, the following histological subtypes were collected: superficial basal cell carcinoma, nodular basal cell carcinoma, sclerodermiform basal cell carcinoma and metatypical/basosquamous cell carcinoma. In addition, ulceration was documented. Diagnostic criteria remained consistent across the study periods.

### Statistical analysis

Data evaluation was performed using SPSS Statistics software (IBM), version 28, and GraphPad Prism, version 8.4.3 (GraphPad Software, Inc.).

In the figures, the data are shown as n/N and percentages (binary variables) with 95% confidence interval or as mean with 95% confidence interval (quantitative variables). Confidence intervals for means were calculated using the t distribution based on the standard error of the mean. For percentages, the two-sided 95% confidence intervals were calculated using the Wilson score method.

Patients assessed at the three time periods represented independent groups. Pairwise comparisons between predefined time periods were performed using Pearson’s chi-square test for categorical variables and two-tailed Mann–Whitney U tests for quantitative variables. Two-sided *P*-values are reported descriptively and no adjustment was applied because analyses were exploratory, hypothesis-generating and restricted to predefined time-period comparisons within each parameter.

A *P*-value of less than 0.05 was considered statistically significant.

## Results

565 patients aged between 18 and 98 years were included in the study. Surprisingly, the number of hospitalized patients per study period increased over time from 147 patients during period 1 (Nov 2019 - Feb 2020) to 199 patients in period 2 (Nov 2020–Feb 2021) and 219 patients in period 3 (Nov 2022 - Feb 2023). Age and gender of all three study groups were similar, with the age of patients (mean ± standard deviation) in period 1 being 75.1 ± 10.8 years (*m* = 50.3%, *w* = 49.7%), 73.1 ± 13.1 years in period 2 (*m* = 50.8 %, *w* = 49.2%) and 73.1 ± 12.1 years in period 3 (*m* = 53.4%, *w* = 46.6%).

**FIGURE 1:**
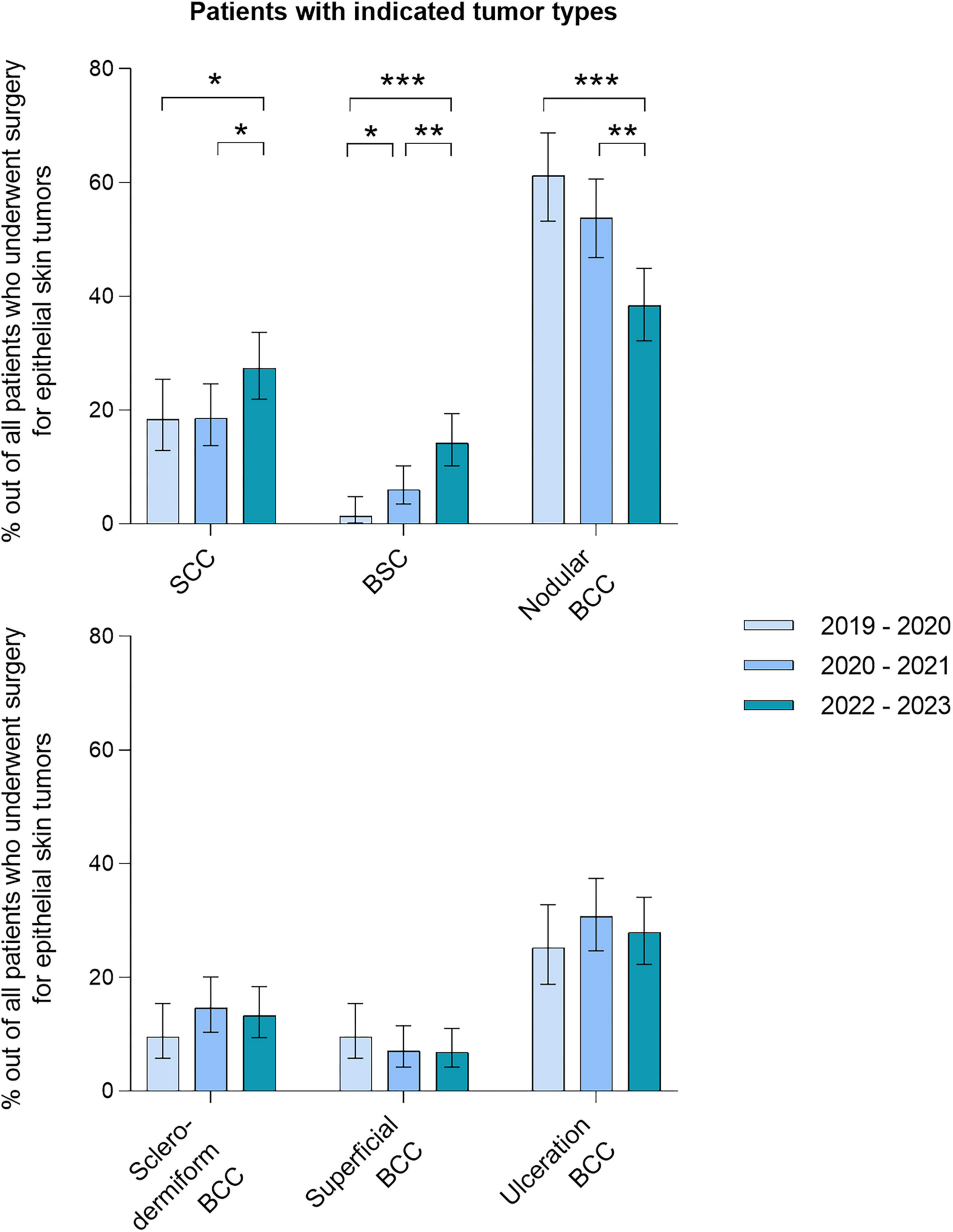
Distribution of tumor types across the observed time intervals. The data were collected retrospectively from the medical hospital records of patients who underwent surgery for epithelial skin tumors during the following three time periods: before (period 1: Nov 2019 –Feb 2020; 147 patients), during (period 2: Nov 2020–Feb 2021; 199 patients) and after the COVID-19 pandemic (period 3: Nov 2022–Feb 2023; 219 patients). Proportions of histopathologically confirmed tumor types among all included patients across the three periods were evaluated. If a patient had more than one tumor, the largest tumor was used for analysis. If the tumor with the highest thickness was ulcerated, this was also noted. The proportions are presented as percentages with two-sided 95% confidence intervals calculated using the Wilson score method. Significance markers: *p < 0.05; **p < 0.01; ***p < 0.001; Statistical analysis performed using chi-square test. SCC, squamous cell carcinoma; BSC, basosquamous basal cell carcinoma; BCC, basal cell carcinoma.

The analysis of NMSC subtypes across the three time periods revealed a gradual increase in the proportion of more malignant tumor entities among patients who had undergone surgery, compared to the pre-pandemic period. In fact, the proportion of patients whose thickest tumor was a squamous cell carcinoma increased significantly from period 1 to 3 (18.4% vs. 27.4%, *n* = 27 vs. *n* = 60, *p* = 0.047) and from period 2 to 3 (18.6% vs. 27.4%, *n* = 37 vs. *n* = 60, *p* = 0.033) (Figure 1). Furthermore, a significant increase was observed in the proportion of patients whose thickest tumor was a basosquamous carcinoma from period 1 to 2 (1.4% vs. 6.0%, *n* = 2 vs. *n* = 12, *p* = 0.029), from period 1 to 3 (1.4% vs. 14.2%, *n* = 2 vs. *n* = 31, *p* < 0.001) and period 2 to 3 (6.0% vs. 14.2%, *n* = 12 vs. *n* = 31, *p* = 0.006) (Figure 1). In addition, a decrease in the proportion of patients whose thickest tumor was a nodular basal cell carcinoma from period 1 to 3 (61.2% vs. 38.4%, *n* = 90 vs. *n* = 84, *p* < 0.001) and from period 2 to 3 (53.8% vs. 38.4%, *n* = 107 vs. *n* = 84, *p* = 0.002) (Figure 1) was noted.

The proportion of patients whose thickest tumor was a sclerodermiform basal cell carcinoma rose (not significantly) from period 1 to 2 from 9.5% to 14.6%, followed by a decline from period 2 to 3 (14.6% to 13.2%). The proportion of patients whose thickest tumor was a superficial basal cell carcinoma showed a general decreasing trend from period 1 to 3. The incidence of ulceration showed an overall rising trend over the study periods, with a slight decline from period 2 to 3 (Figure 1).

**FIGURE 2:**
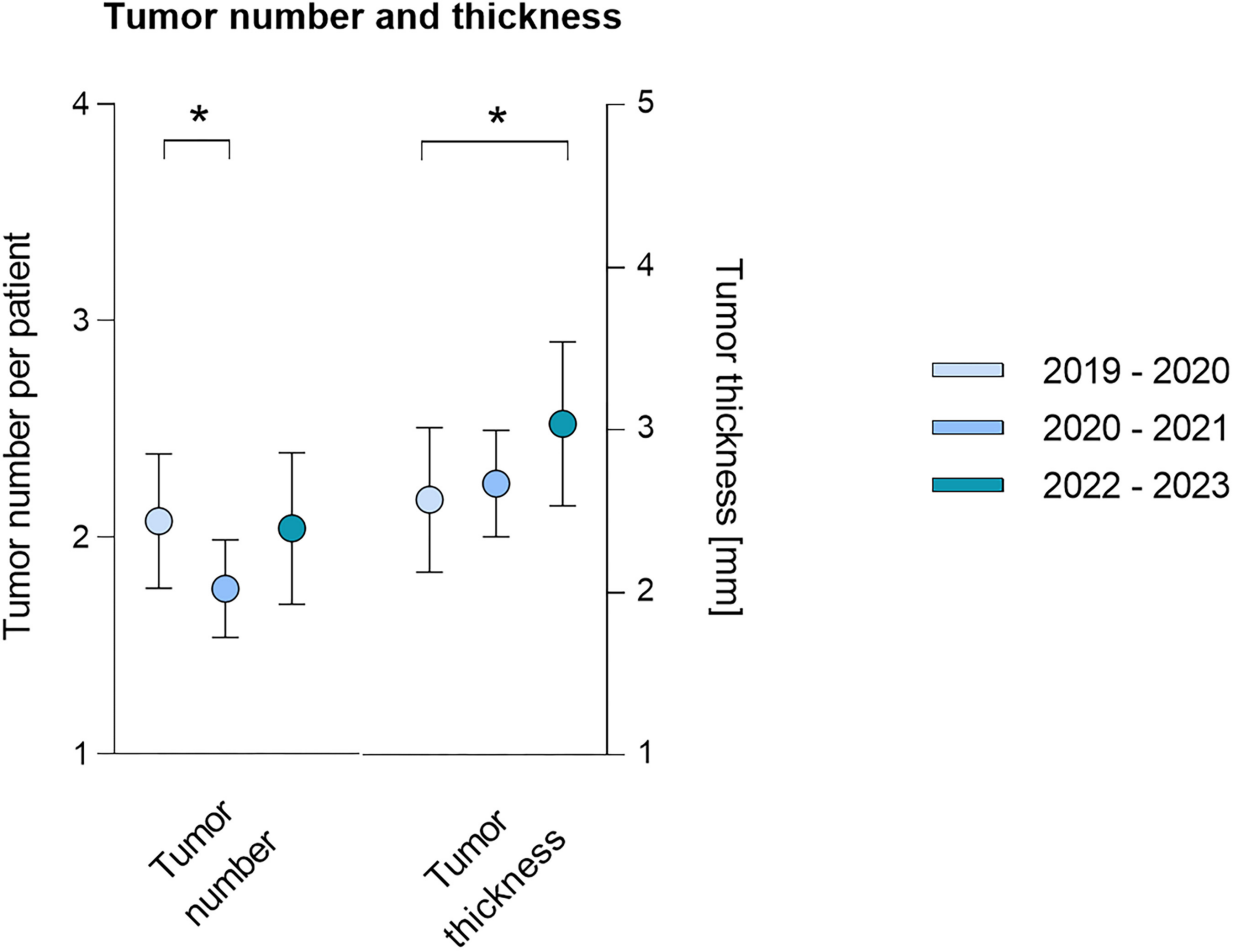
Tumor numbers per patient and tumor thickness across time intervals. The data were collected retrospectively from the medical hospital records of patients who underwent surgery for epithelial skin tumors during the following three time periods: before (period 1: Nov 2019–Feb 2020; 147 patients), during (period 2: Nov 2020–Feb 2021; 199 patients) and after the COVID-19 pandemic (period 3: Nov 2022–Feb 2023; 219 patients). The number of tumors per patient (period 1: 147 patients, period 2: 199 patients, period 3: 219 patients) and measured tumor thickness for the thickest tumor (period 1: 147 patients, period 2: 199 patients, period 3: 217 patients) are shown. The data are presented as the mean + /- the two-sided 95% confidence intervals. Significance markers: *p < 0.05; statistical analysis performed using the Mann–Whitney U test.

**FIGURE 3:**
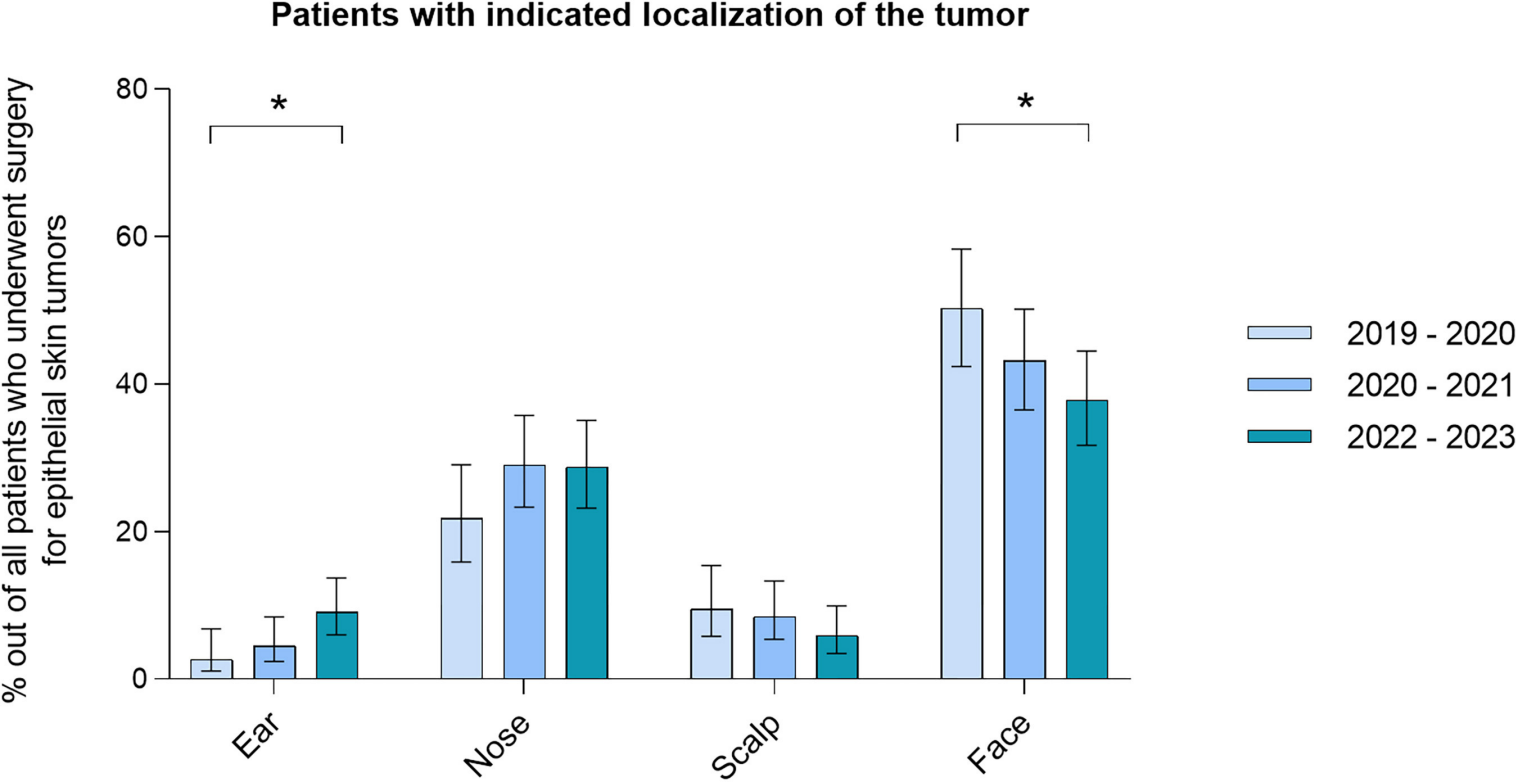
Tumor localization across time intervals. The data were collected retrospectively from the medical hospital records of patients who underwent surgery for epithelial skin tumors during the following three time periods: before (period 1: Nov 2019–Feb 2020; 147 patients), during (period 2: Nov 2020–Feb 2021; 199 patients) and after the COVID-19 pandemic (period 3: Nov 2022–Feb 2023; 219 patients). Anatomical localization of the thickest tumor among all included patients across the three periods were evaluated. The proportions are presented as percentages with two-sided 95% confidence intervals calculated using the Wilson score method. Significance markers: *p < 0.05; statistical analysis performed using chi-square test.

Interestingly, there was a significant decline in the number of tumors per patient (mean ± standard deviation) from period 1 to 2 (*n* = 2.07 ± 1.90 vs. *n* = 1.76 ± 1.60, *p* = 0.046) and a significant rise in tumor thickness (mean ± standard deviation) from period 1 to 3 (2.57 ± 2.73 mm vs. 3.04 ± 3.77 mm, *p* = 0.048) (Figure 2). Furthermore, there was a significant increase in the proportion of patients whose thickest tumor was located on the ear in period 3 (period 1: 2.7%, period 2: 4.5%, period 3: 9.1%; period 1 vs. period 3 *p* = 0.015) and a significant decrease from period 1 to 3 regarding the occurrence in the rest of the face (unspecified localization of the face except nose, ear, scalp) (period 1: 50.3%, period 2: 43.2%, period 3: 37.9%; period 1 vs. period 3 *p* = 0.018) (Figure 3).

**FIGURE 4:**
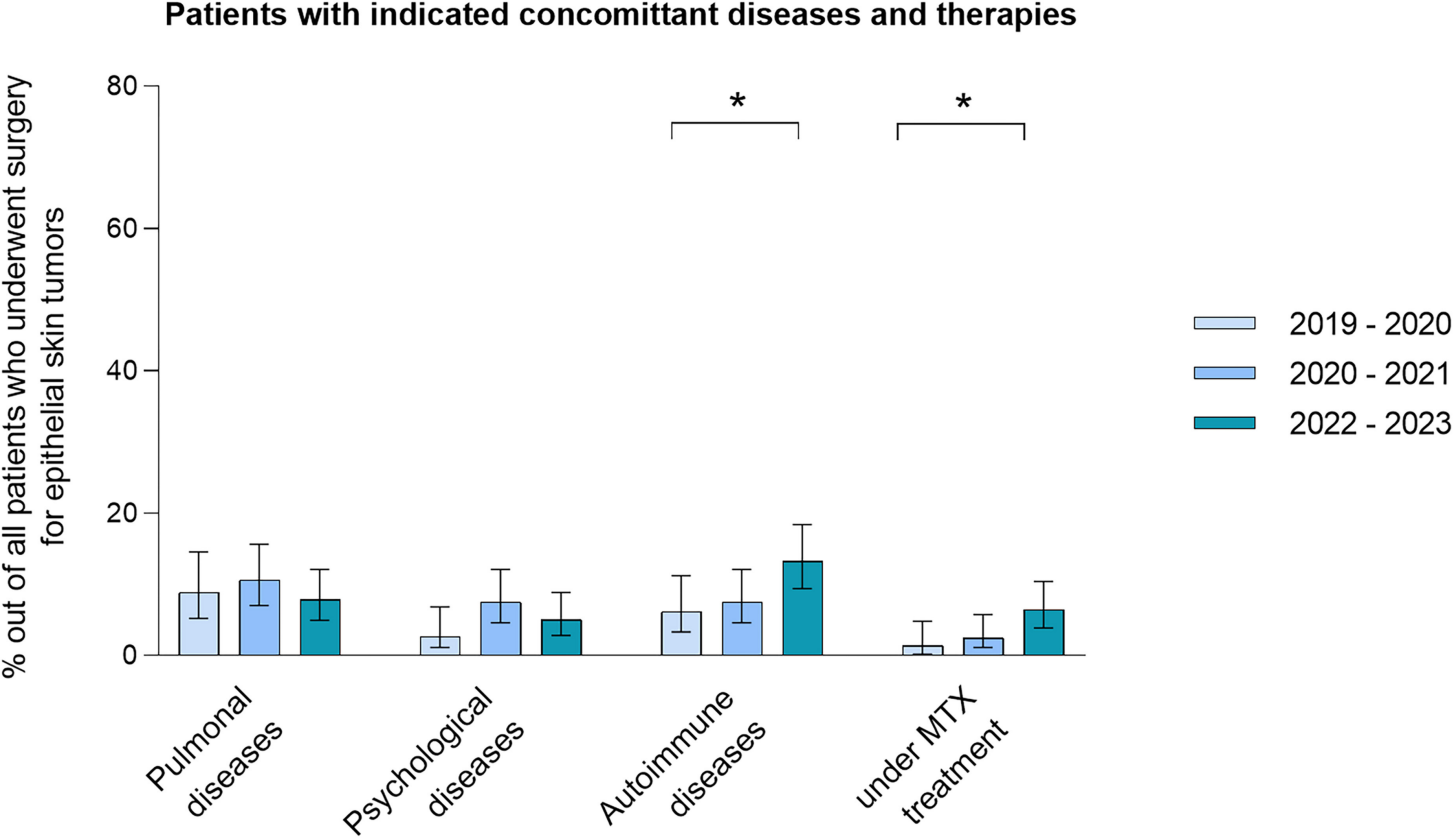
Comorbidities and immunosuppressive treatments across time intervals. The data were collected retrospectively from the medical hospital records of patients who underwent surgery for epithelial skin tumors during the following three time periods: before (period 1: Nov 2019–Feb 2020; 147 patients), during (period 2: Nov 2020–Feb 2021; 199 patients) and after the COVID-19 pandemic (period 3: Nov 2022–Feb 2023; 219 patients). Presence of selected comorbidities and the use of immunosuppressive therapies among all included patients across the three periods were evaluated. The proportions are presented as percentages with two-sided 95% confidence intervals calculated using the Wilson score method. Significance markers: *p < 0.05; statistical analysis performed using chi-square test. MTX, methotrexate.

With regard to the presence of comorbidities, there were significantly more patients who also suffered from autoimmune diseases in period 3 compared to period 1 (period 1: 6.1% vs. period 3: 13.2%, *n* = 9 vs. *n* = 29, *p* = 0.029) and significantly more patients were treated with methotrexate in period 3 than in period 1 (period 1: 1.4% vs. period 3: 6.4%, *n* = 2 vs. *n* = 14, *p* = 0.021) (Figure 4).

**FIGURE 5:**
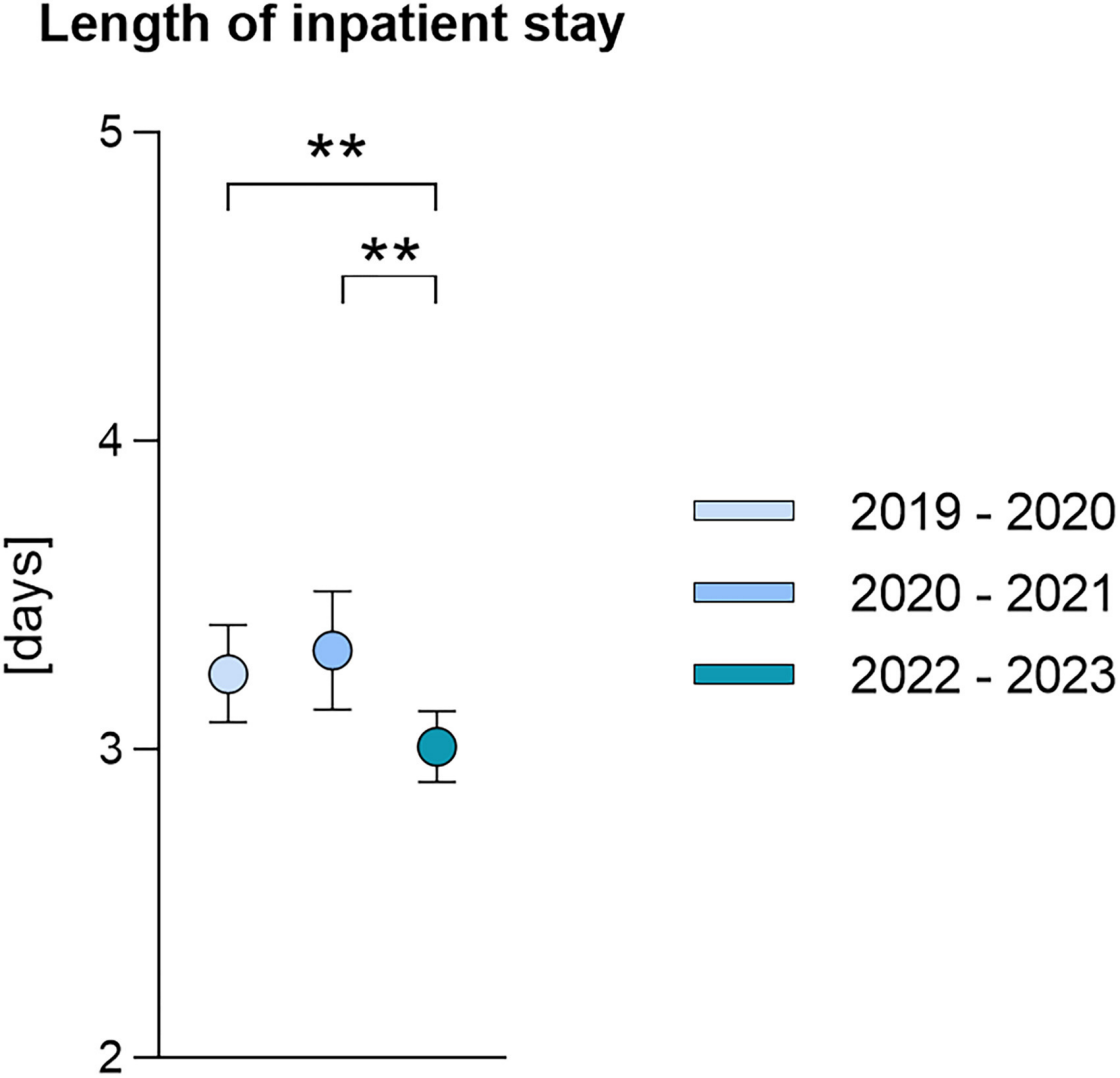
Length of inpatient stay across time intervals. The data were collected retrospectively from the medical hospital records of patients who underwent surgery for epithelial skin tumors during the following three time periods: before (period 1: Nov 2019–Feb 2020; 147 patients), during (period 2: Nov 2020–Feb 2021; 199 patients) and after the COVID-19 pandemic (period 3: Nov 2022–Feb 2023; 219 patients). Duration of inpatient stay among all included patients in the three periods were evaluated. The data are presented as the mean + /- the two-sided 95% confidence intervals. Significance markers: **p < 0.01; statistical analysis performed using the Mann–Whitney U test.

Looking at the length of the inpatient stay, there was a highly significant reduction from period 1 to 3 (3.24 ± 0.96 days vs. 3.01 ± 0.86 days, *p* = 0.003) and from period 2 to 3 (3.32 ± 1.37 days vs. 3.01 ± 0.86 days, *p* = 0.005) (Figure 5).

**FIGURE 6:**
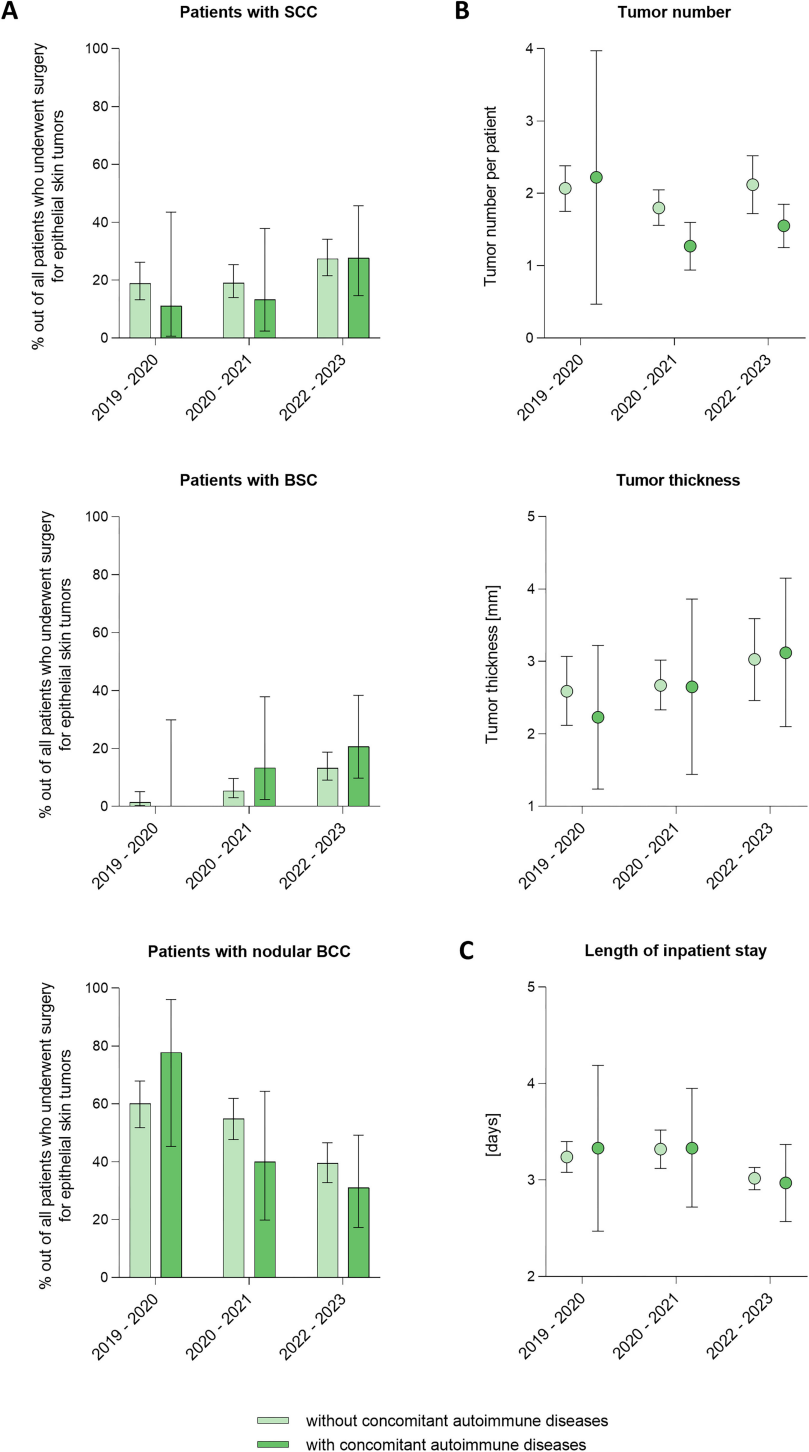
Differences between patients with and without autoimmune diseases regarding epithelial skin tumors. The data were collected retrospectively from the medical hospital records of patients who underwent surgery for epithelial skin tumors during the following three time periods: before (period 1: Nov 2019–Feb 2020; 147 patients), during (period 2: Nov 2020–Feb 2021; 199 patients) and after the COVID-19 pandemic (period 3: Nov 2022–Feb 2023; 219 patients). Patients in each period were divided into two subgroups, depending on whether or not they had an autoimmune disease (AD) (period 1, without AD: 138, with AD: 9; period 2, without AD: 184/182, with AD: 15; period 3, without AD: 190, with AD: 29). **(A)** The proportions of histopathologically confirmed tumor types among all patients were evaluated. If a patient had more than one tumor, the largest tumor was used for analysis. The proportions are presented as percentages with two-sided 95% confidence intervals calculated using the Wilson score method. Statistical analysis performed using chi-square test. **(B)** The number of tumors per patient and the measured tumor thickness for the thickest tumor of each patient were calculated. The data are presented as the mean + /- the two-sided 95% confidence intervals. Statistical analysis performed using the Mann–Whitney U test. **(C)** Duration of inpatient stay for each patient were evaluated. The data are presented as the mean + /- the two-sided 95% confidence intervals. Statistical analysis performed using the Mann–Whitney U test. SCC, squamous cell carcinoma; BSC, basosquamous basal cell carcinoma; BCC, basal cell carcinoma.

Since autoimmune diseases may promote the development of more malignant tumors, we divided the patients from each time period into two groups (those with and those without autoimmune diseases) and examined whether the proportion of NMSC subtypes differed between these two subgroups. As shown in Figure 6A, the proportions of patients whose thickest tumor was a squamous cell carcinoma, basosquamous carcinoma, or nodular basal cell carcinoma did not differ between the two subgroups in any of the analyzed time periods. Furthermore, there were no differences in the number of tumors per patient or in thickness of the thickest tumor between patients with and without autoimmune diseases in any of the analyzed time periods (Figure 6B). The length of inpatient stay was also comparable between the two subgroups during each of the examined time periods (Figure 6C). Although stratification into two subgroups resulted in smaller analyzed populations, we nevertheless observed trends similar to those in the overall population. For example, among patients without autoimmune diseases, the proportion of patients whose thickest tumor was a basosquamous carcinoma increased following the COVID-19 pandemic (period 1 to 3: 1.4% vs. 13.2%, *n* = 2 vs. *n* = 25, *p* < 0.001), whereas the proportion of patients with nodular basal cell carcinoma decreased (period 1 to 3: 60.1% vs. 39.5%, *n* = 83 vs. *n* = 75, *p* < 0.001).

## Discussion

The main findings of our study were changes in tumor characteristics, patient characteristics and length of inpatient stays observed in connection with the COVID-19 pandemic. We found an increase in the proportion of more malignant tumor types as well as an increase in tumor thickness. Furthermore, we found a shortening of inpatient stays and a higher proportion of patients with autoimmune diseases and those receiving methotrexate therapy. We consider the relatively large cohort size, the inclusion of multiple time periods including the post-COVID-19 pandemic period and the unique structure of the healthcare system, which maintained access to dermatological surgery during the pandemic, as strengths of our study. Furthermore, the consideration of both tumor-specific and patient-specific characteristics, as well as reflections on the healthcare system’s preparedness for future pandemics, might present additional strengths of our study.

Regarding tumor characteristics, we observed an increase in the proportion of squamous cell carcinomas and basosquamous carcinomas following the COVID-19 pandemic. Simultaneously, the number of nodular basal cell carcinomas declined among NMSC patients who underwent surgical treatment at our hospital. This trend suggests a shift toward more malignant tumor types. These findings are important because the predominance of more malignant tumor types is associated with a higher probability of more complex surgical procedures, postoperative complications and higher morbidity (13).

Similarly, based on data from 111 patients, Simone Benedetti et al. suggested that limited access to healthcare providers and the prioritization of treating life-threatening conditions had a negative impact on the early diagnosis and management of NMSC in Italy (14). This suggestion was substantiated by James Wall et al., whose data showed a reduction in reported cutaneous squamous cell carcinoma cases in 2020 in 13 countries across Europe. However, numbers increased again by 8% in 2021 compared to 2019, suggesting that many diagnoses were merely delayed and not entirely absent (15).

We also found an increase in tumor thickness following the COVID-19 pandemic among NMSC patients who underwent surgical treatment at our hospital. Although the absolute difference was modest, this may still reflect more advanced tumor growth. However, data on tumor recurrence or patient survival were not available in this retrospective analysis.

Consistent with our results, Silvia Cozzi et al. observed a larger average tumor size and an elevated incidence of cutaneous squamous cell carcinoma in Italy during the COVID-19 pandemic compared to the pre-pandemic period (16). Galina Balakirski et al. also reported an increase in the average tumor thickness in cutaneous squamous cell carcinomas in two German hospitals after the pandemic compared to before (6). Other European studies are consistent with these findings and also show an increase in advanced tumors during the COVID-19 pandemic (8, 16–18). In contrast, a study from the Netherlands, which examined a period from January 2018 to July 2021, concluded that COVID-19-related delays in diagnosis did not lead to the occurrence of unfavorable primary tumor characteristics (tumor thickness of SCC or pT-stage of malignant melanomas) (19). Although the total number of cancer diagnoses (including for skin cancer) declined between March and May 2020 compared to 2019, this decline was largely offset in the fall (20, 21). This may be due to a more rigorous implementation of skin cancer screenings or to less severe restrictions to maintain access to medical care.

In Germany, the COVID-19 pandemic appears to have affected not only the characteristics of the tumors but also the number of skin cancer surgeries performed. In fact, a nationwide analysis of inpatient cases in German hospitals compared data from the pre-pandemic period (March 18, 2019 – March 17, 2020) with data from the first half of the pandemic (March 18, 2020 – March 17, 2021) and found a decline in both surgical procedures and the total number of inpatient skin cancer cases (22). This might be attributed to stricter government regulations in public hospitals under the pandemic emergency plan, which prioritized the care of patients infected with COVID-19. Other studies from Europe also demonstrated a significant reduction in the number of performed skin cancer surgeries, especially regarding malignant melanomas (41%) and squamous cell tumors (44%) during the lockdown in 2020 compared to 2019 (23). Data from the German cancer register also showed a reduction in registered cases of malignant melanomas in Berlin and Brandenburg during the pandemic (24).

The impact of the COVID-19 pandemic on the care of patients with NMSC appears to vary from country to country and depends both on the nature of the restrictions and on the structure of the healthcare system. The study by O. Abbassi et al. from the United Kingdom found that during the first wave of the COVID-19 pandemic, many routine procedures in private practices or smaller facilities were restricted. As a result, more cases were transferred to tertiary centers, where excisions were performed more frequently by experienced specialists. This was accompanied by a reduction in the rate of incomplete excisions as well as a significant increase in NMSC excisions of approximately 160% at the observed tertiary care center compared to 2019. However, this might not reflect a general rise in NMSC cases, but rather a shift from outpatient to inpatient/tertiary care (25).

Our study also detected a shortening of the length of inpatient stays over the entire observation time, with the shortest stays in the post-pandemic period. This shortening might be attributable to structural and organizational factors. During the COVID-19 pandemic, the desire to minimize hospital stays was complemented by a focus on optimizing resource utilization. This was likely made possible by more efficient perioperative management and earlier discharge. However, the shortening of the length of inpatient stays combined with an increase in the severity of cases (more frequent malignant tumors and greater thickness) places a burden on medical staff (26). This trend has also been observed in other international studies (16–18, 23).

In addition, we observed a higher proportion of patients with autoimmune diseases and methotrexate therapy among NMSC patients who underwent surgical treatment at our hospital following the COVID-19 pandemic. Methotrexate is frequently used to treat autoimmune diseases and can lead to chronic immunosuppression, which carries an increased risk of developing NMSC (27–30). The observed increase could indicate a general rise in the prevalence of autoimmune diseases and methotrexate use. This could either be linked to an increased incidence of NMSC among these patients or to a greater proportion of patients being referred to and treated at our hospital after the COVID-19 pandemic.

It is reasonable to assume that the postponement of elective procedures during the COVID-19 pandemic led to delays in the treatment of NMSCs. This resulted in a backlog of cases in the post-pandemic period. We attribute the increase in the number of patients with autoimmune diseases and methotrexate therapy who underwent surgery at our hospital, at least in part, to the fact that chronically ill patients were more likely to seek treatment at a specialized, safer facility even after the COVID-19 pandemic. However, even when considering only patients without autoimmune disease (a subgroup of our cohort), the proportion of patients with basosquamous carcinomas increased following the COVID-19 pandemic, while the proportion of patients with nodular basal cell carcinomas decreased.

The main limitations of this study are its retrospective and single-center design, which can limit the generalizability of the findings. The absence of long-term oncologic outcomes, such as metastasis or survival, and the lack of adjustment for multiple comparisons represent further limitations. Furthermore, changes in referral patterns during and after the COVID-19 pandemic may have affected case selection and thus potentially influenced the composition of the study population. However, most of the changes observed in our study occurred after the COVID-19 pandemic, when most hospitals had returned to normal operations, so bias due to referrals appears rather unlikely. In summary, our findings highlight the need for more specialized healthcare structures to balance pandemic-related care with the continued treatment of critical conditions such as NMSC.

## Conclusion

Our study showed that the COVID-19 pandemic was associated with a subsequent increase in tumor thickness and a higher proportion of patients with more malignant tumor types, among patients with NMSCs who underwent surgery in a hospital without drastic restrictions on care. In addition, the proportion of patients with autoimmune diseases increased, while the duration of inpatient stays shortened. Overall, this places a greater burden on both the healthcare system and patients.

The challenge for similar situations in the future is to ensure timely access to healthcare providers, even when the healthcare system is burdened with new tasks. Especially for vulnerable patient groups, timely diagnosis and early treatment are crucial to prevent delays in care and improve outcomes.

## Data Availability

The data on which the conclusions of this article are based are presented in the figures and in the text. Further data will be made available upon request according to the legal and ethical possibilities by the corresponding author.
